# A Neonatal Murine Model of MRSA Pneumonia

**DOI:** 10.1371/journal.pone.0169273

**Published:** 2017-01-06

**Authors:** Elizabeth A. Fitzpatrick, Dahui You, Bishwas Shrestha, David Siefker, Vivek S. Patel, Nikki Yadav, Sridhar Jaligama, Stephania A. Cormier

**Affiliations:** 1 Department of Microbiology, Immunology and Biochemistry, University of Tennessee Health Science Center, Memphis, TN, United States of America; 2 Department of Pediatrics, University of Tennessee Health Science Center, Memphis, TN, United States of America; University of Georgia, UNITED STATES

## Abstract

Pneumonia due to methicillin-resistant *Staphylococcus aureus* (MRSA) is a significant cause of morbidity and mortality in infants particularly following lower respiratory tract viral infections such as Respiratory Syncytial Virus (RSV). However, the mechanisms by which co-infection of infants by MRSA and RSV cause increased lung pathology are unknown. Because the infant immune system is qualitatively and quantitatively different from adults we developed a model of infant MRSA pneumonia which will allow us to investigate the effects of RSV co-infection on disease severity. We infected neonatal and adult mice with increasing doses of MRSA and demonstrate that neonatal mice have delayed kinetics in clearing the bacteria in comparison to adult mice. There were differences in recruitment of immune cells into the lung following infection. Adult mice exhibited an increase in neutrophil recruitment that coincided with reduced bacterial titers followed by an increase in macrophages. Neonatal mice, however, exhibited an early increase in neutrophils that did not persist despite continued presence of the bacteria. Unlike the adult mice, neonatal mice failed to exhibit an increase in macrophages. Neonates exhibited a decrease in phagocytosis of MRSA suggesting that the decrease in clearance was partially due to deficient phagocytosis of the bacteria. Both neonates and adults responded with an increase in pro-inflammatory cytokines following infection. However, in contrast to the adult mice, neonates did not express constitutive levels of the anti-microbial peptide Reg3γ in the lung. Infection of neonates did not stimulate expression of the co-stimulatory molecule CD86 by dendritic cells and neonates exhibited a diminished T cell response compared to adult mice. Overall, we have developed a neonatal model of MRSA pneumonia that displays a similar delay in bacterial clearance as is observed in the neonatal intensive care unit and will be useful for performing co-infection studies.

## Introduction

Pneumonia due to methicillin-sensitive *Staphylococcus aureus* (MSSA) and methicillin-resistant *Staphylococcus aureus* (MRSA) is a significant cause of morbidity and mortality in infants particularly following lower respiratory tract viral infections such as influenza and Respiratory Syncytial Virus (RSV) [1–3]. In reviews of invasive or respiratory community-onset *S*. *aureus* infection during influenza season, the proportion of RSV coinfection is similar to that for influenza coinfection [1, 4]. Identifying the pathogenic mechanisms that lead to the increased mortality in MRSA-induced pneumonia in the presence of viral co-infections is necessary for the identification of new therapeutic targets to treat the disease.

In the US, the most common strain of MRSA is USA300 which is more virulent than MSSA and there is evidence of new MRSA strains developing that have increased resistance to antibiotics[5] [6]. Initially, MRSA was considered a hospital-acquired pathogen however there has been a global increase in the incidence of community-acquired MRSA (CA-MRSA) in both adults and children [2, 7]. Infants in the neonatal intensive care unit (NICU) developed MRSA pneumonia at a younger age, had a lower birth weight and a longer duration of positive cultures than infants with MSSA [8, 9]. MRSA pneumonia in adults is characterized by a strong inflammatory response including a neutrophilic influx into the lung, pulmonary edema, loss of alveolar architecture and hemorrhage [10, 11]. The pathology caused by MRSA is due to the many virulence factors produced by the bacteria as well as to the overwhelming host immune response to the infection. MRSA possesses the same virulence factors that contribute to pneumonia as MSSA such as the toxins Panton-Valentine Leukocidin (PVL), α-hemolysin and protein A (SpA) [12, 13]. However, the host immune response also contributes to lung damage observed during pneumonia. For example, alpha-hemolysin is a pore forming protein that activates the inflammasome causing IL-1β and IL-18 production and an increase in pyroptosis, an inflammatory form of cell death [14–16]. MRSA also possesses several toxins with superantigen properties such as Toxic shock syndrome toxin (TSST-1) and enterotoxins A and B. These toxins induce a robust inflammatory cytokine response by crosslinking TcRs with MHC II on antigen presenting cells [17, 18]. The role of MSSA and MRSA in influenza has been characterized and recent data suggest RSV contributes to *S*. *aureus* pneumonia [1]. Initial infection with RSV occurs mostly during infancy and there are significant differences between the adult and neonatal immune response, so before we could address the role of RSV in contributing to *S*. *aureus* pneumonia, we had to create a neonatal mouse model of MRSA.

Neonates are more susceptible to microbial infections, which is thought to be due to deficiencies in the immune response compared to adults. In most cases, the deficiencies reflect a quantitative reduction in the level of the immune response. The neonatal complement system has reduced opsonizing activity and lytic activity [19]. Neonatal neutrophils exhibit impaired migration, reduced anti-microbial peptide production and newborns that developed sepsis exhibit a suppressed respiratory burst [20, 21]. Soluble factors such as adenosine, which inhibits TLR-mediated TNF-α production, are increased in neonatal plasma and may also contribute to an overall anti-inflammatory environment [22, 23]. However, there are also distinct differences in the immune response generated by neonates and adults in response to microbes. Pattern recognition receptor stimulation of neonatal innate immune cells results in a distinct profile of cytokines compared to adult cells. For instance following TLR stimulation, neonatal cells produced lower levels of IL-12p70, IFNα and IFNγ and higher levels of IL-23, IL-6, IL-1β and IL-10 compared to adult cells [22]. The decrease in pro-inflammatory cytokines and increase in anti-inflammatory cytokines such as IL-10 contributes to neonatal susceptibility to microbial infections [24, 25]. Additionally, the altered cytokine response produced by neonatal innate immune cells results in differences in the adaptive immune response. Evidence suggests that in neonates there is preferential differentiation of naïve CD4^+^ T cells into Th2 or Th17 subsets [22, 26–28]. The Th1 response is delayed which is likely related to the decreased IL-12p70 levels [29]. The mechanism(s) by which infants respond to MRSA pneumonia are unknown and because the host immune response contributes to lung pathology it is unclear whether the anti-inflammatory environment of the infant lung in the face of reduced neutrophil function affords them some protection. Because of the increasing incidence of community-acquired pneumonia due to MRSA in infants, it is imperative to identify the pathogenic mechanisms that contribute to disease.

The objective of this study was to define the neonatal mouse response to MRSA and identify the immunologic factors that contribute to or protect against lung pathology in the disease in order to gain insight into disease mechanism in human infants.

## Materials and Methods

### Animals and MRSA infection protocol

C57BL/6 female and male mice were purchased from Jackson Laboratories (Bar Harbor, ME) at 6 weeks of age to set up as breeders. The breeders were time-mated and pups born on the same date were used for experiments. A set of pups were allowed to mature to 6–8 wks of age, and were then used as adult controls. For all infections neonatal mice were used at 5 or 6 d old. For survival studies mice were monitored every 12 hours and assigned a clinical score using a scale of 0–5. Criteria used to assign the score were: 0 = healthy, 1 = barely ruffled fur, 2 = ruffled fur, 3 = ruffled fur and inactive, 4 = ruffled fur, inactive, gaunt, 5 = dead. For humane reasons mice that reached a clinical score of 4 were euthanized. The neonatal mice infected with the highest dose of MRSA all died without euthanasia before the first monitoring period was up; this was unavoidable to set infectious dose, since there have been no MRSA neonatal pneumonia models published to provide guidance in determining our dose range. Because an overwhelming immune response contributes to lung pathology and neonatal mice have deficient immune responses, it was conceivable that the highest dose used (which was sublethal in adults) would also be sublethal in the neonates. This data provided us with the necessary information to choose a sublethal MRSA dose for the neonatal mice that was used for the remainder of the studies. All animals were housed in sterile micro-isolator cages with sterile food and water ad libitum and were maintained by the Division of Comparative Medicine at the University of Tennessee Health Science Center according to the guidelines of the Animal Welfare Act. All animal care procedures were performed according to protocols approved by the UTHSC animal care and use committee.

MRSA (USA300; gift from Jay Kolls) was added to Trypticase Soy Broth (TSB; BD) and incubated overnight at 37°C with shaking. An aliquot of this suspension was used to prepare a 1/100 dilution in TSB which was incubated for 2.5 hr at 37C. Aliquots were washed and frozen at -80°C; for mouse infections frozen aliquots were thawed and diluted in 0.9% saline and mice immediately infected. Unless otherwise indicated mice were infected intranasally with between 1.0 x 10^7^–2.0x10^7^ CFU’s in a total volume of 10 μl for neonates or 50 μl for adults.

### Lung bacterial clearance

Lungs (both right and left) were harvested at 1, 2 and 3 days post-infection (dpi) and homogenized in 1ml PBS. Serial dilutions were prepared in PBS and plated on Trypticase Soy agar plates. Following 24 hr incubation, the bacterial colonies were counted and colony forming units (CFU) /ml of lung homogenate was calculated.

### Bronchoalveolar lavage (BAL) and lung cell suspension

BAL was performed by intratracheal injection of PBS into the lungs with immediate vacuum aspiration. Cells were recovered from the BAL fluid by centrifugation and counted using trypan blue dye exclusion. The BAL fluid (BALF) was frozen at -80°C until used for ELISA assays for cytokine and chemokine measurement. Lungs were perfused with PBS, removed and disassociated with the gentleMACS™ Octo Dissociator (Miltenyi Biotech) and incubated at 37°C for 30 minutes in HBSS (HyClone) containing 1 mg/ml collagenase I (Invitrogen), and 150 μg/ml DNase I (Sigma-Aldrich). After incubation, the suspension was passed through a 40-μm cell strainer (BD Biosciences) to obtain a single lung cell suspension. Red blood cells were lysed using RBC lysis buffer (eBioscience) and cells were then suspended in RPMI1640 media (HyClone) supplemented by 5% heat-inactivated FBS (HyClone), 100 U/ml penicillin, 100 mg/ml streptomycin (HyClone) and used for surface or intracellular staining.

### Differential cell counting

Slides were air-dried and stained with a modified Wright Giemsa stain (HEMA 3 stat pack, Fisher Scientific) according to manufacturer’s instructions. Three hundred cells were counted and differentiated on each slide by two unbiased observers using standard morphological criteria.

### Cell surface and intracellular flow cytometry

To determine the CD4^+^ T cell populations in the lung, single cells were isolated as above and stimulated for 5 h in RPMI-1640 (HyClone) containing 10% heat inactivated fetal bovine serum (FBS; Life Technologies), 100 U/ml penicillin (HyClone), 100 mg/ml streptomycin (HyClone), 5 ng/ml phorbol-12-myristate-13-acetate (PMA; Sigma-Aldrich), and 500 ng/ml ionomycin (Sigma-Aldrich) in the presence of a protein transport inhibitor (1 μl/106 cells; GolgiPlug, BD Biosciences, Franklin Lakes, NJ). After stimulation, the cells were stained with fixable viability dye, fixed and permeabilized, and labeled with antibodies to CD3 (17A2), CD4 (RM4-4; Biolegend), IFNγ (XMG1.2), IL-4 (BVD6-24G2) and IL-17 (17B7; eBioscience). Flow data were then acquired on a Canto II flow cytometer (BD Biosciences) and analyzed and plotted with FlowJo software (v10 for Windows; Tree Star; Ashland, OR, USA). All antibodies and viability dye were from eBioscience (San Diego, CA, USA) unless otherwise stated.

To determine the myeloid dendritic cell (mDCs) responses in the lung, single cells were isolated as aforementioned and stained by antibodies against CD11c (N418), MHCII (M5/114.15.2), CD80 (16-10A1), and CD86 (GL1). Flow data were then acquired, analyzed and plotted as above.

### RNA isolation and qRT-PCR

Total RNA was extracted from the both lungs of individual mice using RNeasy Plus Universal Kit (Qiagen) according to manufacturers’ directions. Two μg of RNA was reverse transcribed into cDNA with the Superscript III First Strand Synthesis Kit (Thermo Fisher). Real time PCR was performed with gene specific primers to IL-17A, IFNγ, IL-6, TNFα, Reg3γ and Reg3β (S1 Table) using a LightCycler 480 real time PCR thermal cycler (Roche Diagnostics, Indianapolis, IN). All primers and probes were chosen using the online software Universal Probe Library. Data are normalized for HPRT and plotted as relative gene expression.

### In vivo phagocytosis assay

To measure the amount of phagocytosis, bacteria was labeled with the fluorescent dye TAMRA (Life Technologies, NY) by incubating MRSA suspended in PBS with TAMRA dye (12.5μg/ml) on ice for 10min in the dark followed by two washes with PBS to remove the excess dye. Adult and neonatal mice were intranasally inoculated with 2.0 x 10^7^ CFU’s of TAMRA-MRSA or unlabeled MRSA as a control. Whole lungs were removed at 30 min post-infection and a single cell suspension was prepared as described above. The cells were fixed with 4% paraformaldehyde and extracellular fluorescence was quenched using 0.4% trypan blue immediately prior to running on the flow cytometer. The percent of lung cells that expressed TAMRA labeled MRSA was measured using cells from mice exposed to unlabeled MRSA as the gating control.

### ELISA

Lungs were removed and weighed prior to being snap frozen and stored at -80C until used for analysis. The lungs were homogenized in T-PER® (5X weight; Thermo Scientific) containing protease inhibitor cocktail and centrifuged at 10,000 x g for 5min. The supernatants were collected and total protein concentration determined using Bio-Rad protein assay. Cytokines present in lung homogenates were measured by ELISA according to manufacturer’s instructions (Biolegend, San Diego, CA). Cytokine standards ranging from 15.6 pg/ml to 1,000 pg/ml were prepared to determine the concentration of cytokine in the samples. For data analysis, a curve fit was applied to the standards and the sample concentrations extrapolated from the standard curve using four-parameter logistic software (SoftMax Pro, Sunnyvale, CA).

### Statistics

Figures were prepared and statistical analysis performed using GraphPad Prism statistical software (GraphPad Software, San Diego, CA). Survival studies were analyzed using the log rank Mantel-Cox test to determine significant differences in survival between infected neonates and infected adult mice at each dose. The multiple t-test with Holmes-Sidak correction was used to evaluate significance for studies involving bacterial load, cellular composition of BAL fluid and cytokine measurements. The phagocytosis data was analyzed using an unpaired two-tailed t test. Intracellular cytokine staining data were analyzed using one-way ANOVA with Sidak’s correction and two-way ANOVA with Tukey correction was used for analysis of the DC flow cytometry data Data are represented as mean ± SEM and differences were considered significant at p values of less than 0.05.

## Results

### Neonatal mice succumbed to high-dose MRSA infection and were delayed in clearing MRSA with low-dose infection

**Fig 1:**
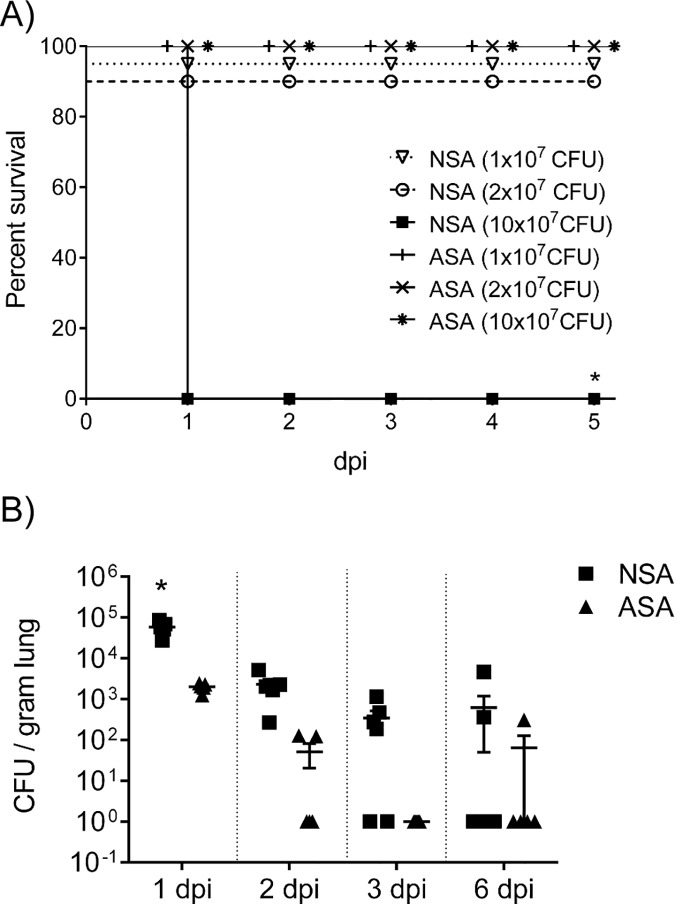
Survival and bacterial clearance of neonatal mice to MRSA. (A) C57Bl/6 neonate (5 d) and adult mice (6 wks) were intranasally infected with increasing doses of USA300 (6 mice/group) and survival was determined over a 6 d period. (B) C57Bl/6 pups (5 d) and adult mice (6 wks) were intranasally infected with 1x10^7^ CFU’s of MRSA and the level of bacteria in the lung was measured on 1, 2 3 and 6 dpi. NSA = neonatal / MRSA infection, ASA = adult / MRSA infection.

To determine the lethal and sublethal doses of MRSA for neonates, we infected neonates (NSA) and adult (ASA) mice intranasally with 1 x 10^7^, 2 x 10^7^ and 10 x 10^7^ CFU’s and measured survival days post-infection (dpi) (Fig 1A). The results demonstrate that neonates infected with 10 x 10^7^ CFUs succumbed to infection by 1 dpi, whereas none of the neonates infected with the lower doses or any of the adult mice died. Because we wanted to investigate the neonatal immune response to MRSA in the absence of mortality and an overwhelming neutrophil response and its subsequent pathology, we performed the remainder of the experiments with 1.0–2.0 x 10^7^ CFUs / mouse. We chose to use the same dose in the adults because our goal is to determine if there are qualitative rather than quantitative differences between the neonatal and adult immune response to MRSA. Additionally, using the sublethal dose is more likely to mimic natural exposures in contrast to models using much higher doses.

We examined bacterial clearance by neonates and adults following MRSA infection at 1 and 3 dpi (Fig 1B). The majority of the adult mice cleared the bacteria within 3 days following infection with 70% of mice clearing the bacteria by 2 dpi; although 1 mouse had detectable MRSA in the lungs at 6 dpi. In contrast, neonatal mice were delayed in clearing the bacteria; by 3 dpi only 2 of the 6 neonatal mice cleared the bacteria and the bacterial load in the remaining mice was not significantly reduced compared to 2 dpi. By 6 dpi, all but 2 of the 8 infected neonatal mice cleared the bacteria. These results suggest that a sublethal dose of MRSA in neonates is very slowly cleared from the lungs.

### Neonatal mice have a decreased inflammatory response following MRSA infection

**Fig 2:**
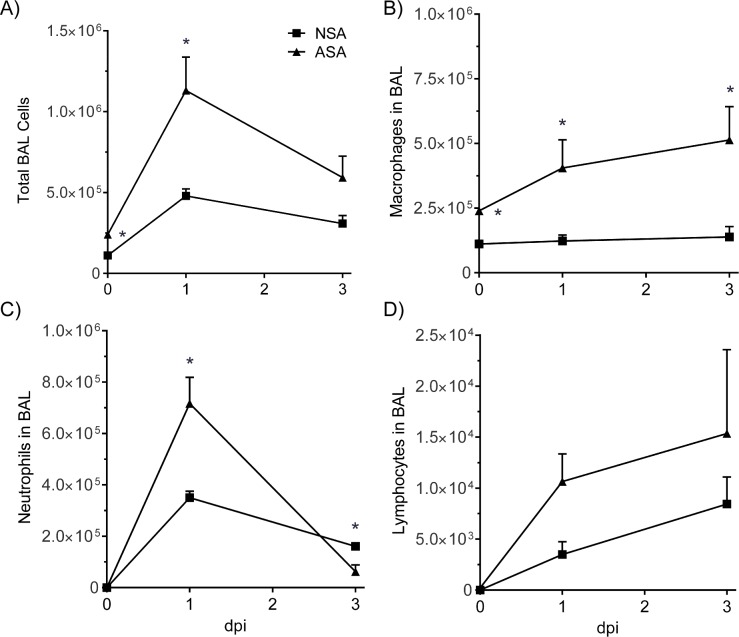
Immune cell recruitment into the lungs of neonatal and adult mice infected with MRSA. Neonatal (5 d) and adult mice (6 wks) were infected with 1x10^7^ CFU’s of MRSA and BAL performed at 1 and 3 dpi. The cells recovered from the BALF were counted by differential staining. NSA = neonatal / MRSA infection, ASA = adult / MRSA infection.

The slower kinetics of bacterial clearance in the neonatal mice may be due to several factors including a reduced neutrophil influx following infection. To investigate this, we measured the extent and cellular composition of BALF in neonates and adults at 1 and 3 dpi (Fig 2). The results demonstrate that there were similar fold increases in total cells recovered from the BALF of both adults and neonates which peaked at 1 dpi and then began to decline through 3 dpi in both age groups. Neutrophils were the predominant cell type in the BALF at 1 dpi for both the adult and neonatal mice. By 3 dpi the neutrophil levels in the MRSA infected adult mice had dropped almost to the level of the control mice, which corresponded with the decline in bacterial load. The neutrophil levels in the MRSA infected neonatal mice also began to decline after 1 dpi although not to control levels and despite the continued presence of bacteria in the lungs. The macrophage response differed between the neonate and adult mice following MRSA infection. After the initial drop in macrophage numbers at 24 hr pi in both neonatal and adult mice, the adult mice exhibited a steady increase in macrophage numbers through 3 dpi. Whereas there was only a marginal increase in macrophages in the lungs of neonatal mice between 1 and 3 dpi. There were no significant changes in the numbers of lymphocytes or eosinophils between the groups. These results suggest that there are differences in inflammatory cell recruitment between adult and neonatal mice following MRSA infection.

### Neonatal lungs cells are less efficient in phagocytosis of bacteria

**Fig 3:**
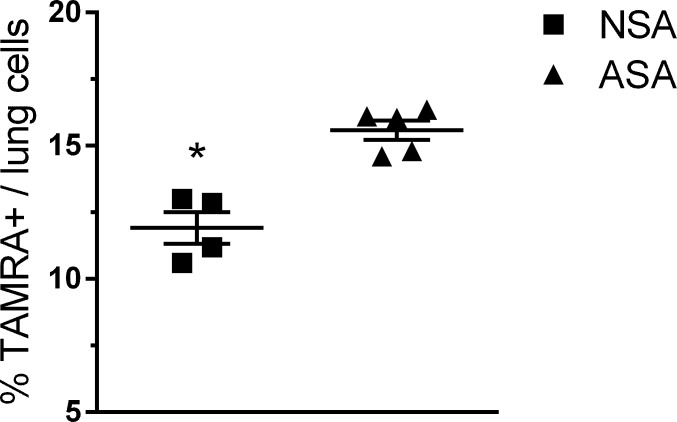
In vivo phagocytosis of MRSA by neonatal and adult mice. Neonate and adult mice were infected with 2x10^7^ CFU’s of TAMRA-MRSA or unlabeled MRSA. The lungs were removed 30 min pi and intracellular expression of TAMRA was measured by flow cytometry. The level of background fluorescence measured using unlabeled MRSA was 6.7%. NSA = neonatal / MRSA infection, ASA = adult / MRSA infection.

It has been previously reported that neonatal macrophages are deficient in their ability to phagocytize bacteria and this may contribute to the slow clearance of MRSA by the neonates. However, isolating a pure population of macrophages from 5 d old mice is difficult due to the small number of alveolar macrophages recovered from the BALF and the decreased adherence of neonatal macrophages compared to adult macrophages. Therefore, we measured in vivo phagocytosis of MRSA in whole lungs from adult and neonatal mice. We infected adult and neonatal mice with TAMRA-labeled MRSA and measured the uptake of the labeled MRSA by lung cells at 30 min pi. This time point was chosen because it precedes the neutrophil influx and likely represents phagocytosis by alveolar macrophages (NSA = 99.3% macrophages, 0.6% neutrophils; ASA = 99.6% macrophages, 0.2% neutrophils, 0.1% lymphocyte; n = 3-4mice / group). Phagocytosis was measured by flow cytometry and the fluorescence emitted by extracellular bacteria was quenched with trypan blue (Fig 3). The results demonstrate that a decrease in TAMRA^+^ cells in the lungs of neonatal mice compared to adult mice. This data suggests that while neonatal mice are capable of bacterial phagocytosis, they are also less efficient at it compared to adult mice.

### Neonatal mice exhibit a pro-inflammatory cytokine profile but altered anti-microbial peptide production following MRSA infection

**Fig 4:**
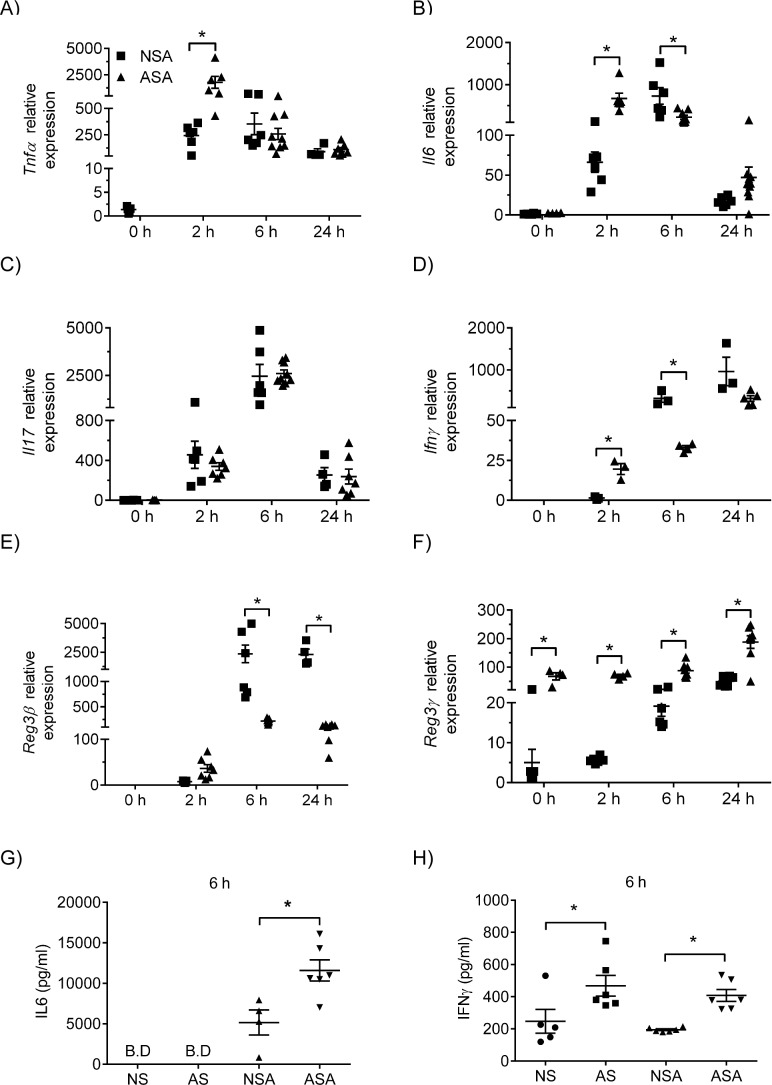
Proinflammatory cytokine and anti-microbial peptide expression in neonatal and adult mice. Neonate and adult mice were infected with 1x10^7^ CFU’s of MRSA and cytokine gene expression measured at 6 and 24 hr pi (A-F). Expression of mRNA for cytokines and anti-microbial peptides was determined by qRT-PCR on RNA isolated from individual lung lobes (n = 5–6 mice per group) and relative expression is fold induction over WT unexposed mice of the same age. The level of IL6 (G)and IFNγ (H) in the lung homogenates was measured by ELISA. The data represent mean ± SEM of duplicate samples and significance was determined using one-way ANOVA with Tukey post-hoc test. NSA = neonatal / MRSA infection, NS = Neonatal / sham infection, ASA = adult / MRSA infection, AS = Adult sham infection.

Numerous studies have demonstrated altered patterns of cytokine production by neonatal cells in response to TLR stimulation compared to adult cells. In particular, the cytokine profile of neonates favors an anti-inflammatory environment with increased IL10 and decreased TNFα and IFNγ production. To determine if there were qualitative differences in expression of proinflammatory cytokines, qRT-PCR was performed on lungs from neonate and adult mice at 6 and 24 hr post-infection (Fig 4). There were quantitative and kinetic differences between neonates and adults. The results demonstrate an increase in the relative expression of mRNA for Tnfα, IL6, and IL17A at 2 hr pi which decreased by 24 hr pi in both the neonatal and adult mice. The increase in Tnfα and IL6 mRNA expression at 2 hr pi was greater in the adult mice compared to the neonates. Similarly, the level of IL6 protein in the lungs at 6 hr pi was greater in the adult mice than the neonates. In contrast, Ifnγ mRNA expression in both neonates and adults was detectable at 2hrs and continued to increase through 24 hr pi in both groups of mice. These results suggest that neonates develop a strong pro-inflammatory response to MRSA despite their immature immune system.

Several studies have demonstrated that neonates have a decrease in the amount of anti-microbial peptides in the plasma which may contribute to decreased microbial resistance. Choi et al [30] demonstrated that Reg3γ is critical to host defense against MRSA using a mouse MRSA pneumonia model. We used qRT-PCR to measure the level of Reg3γ and Reg3β in the neonate and adult mice. Our results demonstrate that adult mice have a basal level of Reg3γ mRNA that is absent in the neonates. Following infection, both neonate and adult mice exhibited an increase in Reg3γ at 6 hr pi which continued to increase through 24 hr. In contrast, both neonates and adults exhibited an increase in Reg3β mRNA expression at 2 hr pi which continued to increase at 6 and 24 hr, although to a greater extent in the neonates.

### Neonatal mice develop limited T helper cell response following MRSA infection

**Fig 5:**
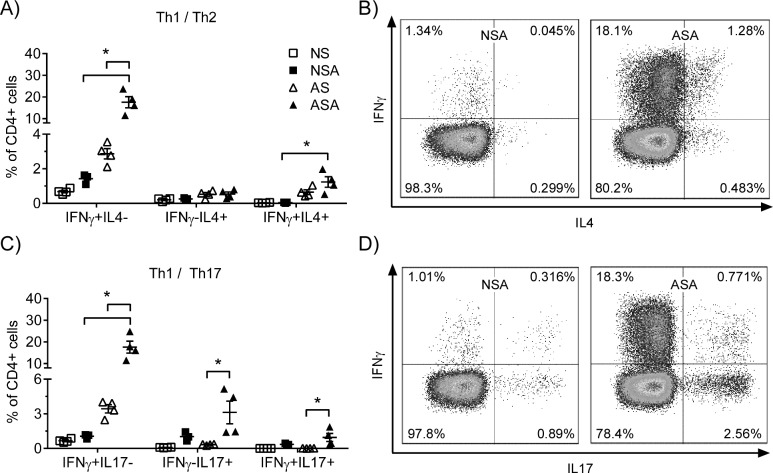
Neonatal mice mounted limited Th1 and Th17 response to MRSA infection. Neonate and adult mice were infected with 1.24 x10^7^ CFU’s of MRSA or vehicle. Lung single cells were isolated at 6 dpi and Th profile was measured by flow cytometry. (A) Th1/Th2 cell percentage of CD4+ T cells. (B) Representative flow plots of Th1/Th2 percentage of CD4+ T cells. (C) Th1/Th17 cell subsets. (D) Representative flow plots of Th1/Th17 subsets. NSA = neonatal / MRSA infection, NS = Neonatal / sham infection, ASA = adult / MRSA infection, AS = Adult sham infection.

Infants with MRSA pneumonia have a delay in bacterial clearance compared to adults. We observed a similar delay in bacterial clearance in our neonatal mouse model. Therefore, although it is doubtful that T cells contribute to MRSA clearance in the adults, they may contribute to clearance in neonates providing an appropriate Th response develops. Th17 responses are necessary for host defense against extracellular bacteria at mucosal surfaces. However, there have been mixed reports in the literature on the extent to which neonates are biased towards a Th2 response or a Th17 response. To measure the CD4^+^ T cell response to MRSA by neonates, we infected neonatal and adult mice with MRSA and measured the T cell response in the lungs at 7 dpi (Fig 5). The results demonstrate that adult mice have a significant increase in the frequency of both Th1 (IFNγ^+^/IL-17^-^) and Th17 (IFNγ^-^/IL-17^+^) cells following MRSA infection with little or no increase in Th2 cells or Tregs (data not shown). In contrast, there was no statistically significant difference in the frequency of Th1 or Th17 cells in infected or uninfected neonatal mice. These results suggest that neonatal mice have a Th cell response that is significantly diminished in comparison to the adult mice.

### Dendritic cells in adult mice develop an increase in co-stimulatory molecule expression

**Fig 6:**
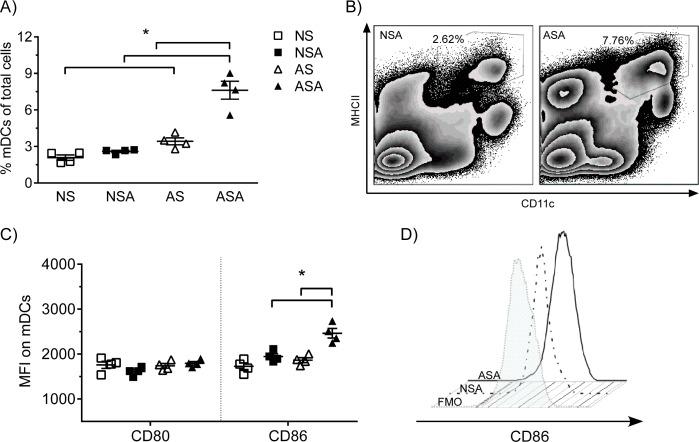
Limited recruitment and maturation of mDCs to the lungs in response to MRSA infection. Neonate and adult mice were infected with 1.24 x10^7^ CFU’s of MRSA or vehicle. Lung single cells were isolated at 6 dpi and the amount of mDCs and their maturation status were measured by flow cytometry. (A) The frequency of mDCs in the lungs as a percentage of total lung cells. (B) Representative flow plots of mDCs. (C) Maturation status of mDCs in the lung. (D) Histogram of CD86 on mDCs. FMO = fluorescence minus one and is used to identify and gate cells in the complex fluorescent dye panel. NSA = neonatal / MRSA infection, NS = Neonatal / sham infection, ASA = adult / MRSA infection, AS = Adult sham infection.

The lack of a significant T cell response to MRSA in the neonatal mice may be due to a decrease in activation and maturation of dendritic cells (DCs). To determine if the mDCs (CD11c^+^/MHCII^+^) are activated in the neonatal mice, we measured the frequency of mDCs in the lung and the maturation markers, CD80 and CD86, on these cells (Fig 6). The results demonstrate that neonatal mice induced very limited mDC response in the lungs, whereas adult mice recruited substantial mDCs into the lungs at 7 dpi (Fig 6A). In addition, there was also an increase in CD86 expression on the mDCs in the adult mice, whereas no significant increase of CD86 on mDCs was observed in neonatal mice (Fig 6B). The expression of CD80 on mDCs was not altered due to infection in either the neonates or adults. These results suggest that the lack of a CD4^+^ T cell response may be due to the insufficient mDC activation.

## Discussion

There has been an increase in the number of cases of MRSA pneumonia in infants due to the increased spread of community-associated USA300 [4]. This is significant because of the increased mortality rate, as well as increased hospital costs associated with MRSA pneumonia in particular following viral infection [2]. However, the pathogenic mechanisms leading to worsening outcome in infants following co-infection are unknown and it is critical that we identify factors contributing to it. In this study we developed a model of infant MRSA pneumonia in neonatal mice that will be important for the study of viral-bacterial coinfections in infancy.

Infection of neonatal mice with MRSA revealed that they were slow to clear MRSA from the lung; at 3 dpi less than half of the neonatal mice had cleared the bacteria and the decrease in bacterial counts was not significantly different from 2 dpi. One explanation for this may be that MRSA is able to survive intracellularly within neonatal neutrophils or macrophages better than in adult cells. We did not test this directly; however, we did not lyse the cells contained in the lung homogenate when performing the bacterial clearance assay. Therefore, the bacteria remaining in the lung likely represent extracellular bacteria that has not been phagocytized or killed by anti-microbial peptides in the neonates. There is evidence of delayed clearance of organisms in other neonatal pneumonia models. Kurkjian et al [31] demonstrated that neonatal mice are delayed in the clearance of the fungal pathogen *Pneumocystis carinii* and this was related to a deficient pro-inflammatory response and increased IL10 and TGFβ in the neonatal lung. Our results also mimic observations in the NICU that neonates with MRSA pneumonia exhibit positive cultures for longer periods of time than MSSA suggesting that there is delayed clearance of MRSA in human neonates also [8, 9].

Neutrophils are critical for efficient clearance of MRSA and they were recruited into the lungs of neonatal mice with the same kinetics as adult mice; peaking at 1 dpi. In the adult mice the drop in neutrophils corresponds with the clearance of the bacteria; in contrast the neutrophil influx in the neonates also began to decline despite the continued presence of bacteria in the lung. This may be due to the limited capacity of neonatal bone marrow to increase neutrophil production as compared to adult bone marrow [32]. Because we used the same dose of MRSA for the adult and neonatal mice it is difficult to do a quantitative comparison, however the fact that the recruited neutrophils do not completely clear the bacteria may reflect decreased phagocytic or killing activity by the neonatal neutrophils. Deficient killing of bacteria by neonatal neutrophils has been demonstrated in several models of sepsis and pneumonia [33, 34]. There have also been reports that macrophages in neonates are deficient in phagocytic activity [33, 35]; our in vivo phagocytosis studies indicated that although neonatal cells were able to phagocytize some TAMRA labeled MRSA, there was a significantly decreased % of TAMRA^+^ cells in the neonates compared to adult mice. We could not perform cell surface fluorescent labeling with macrophage specific markers due to the use of trypan blue to quench fluorescence from extracellular bacteria. The early time point strongly suggests that the TAMRA^+^ cells are macrophages and not neutrophils, since neutrophils have not entered the lung in significant numbers at this time. The deficiency in phagocytosis in the neonatal mice may contribute to the failure to clear the bacteria. Because the neonatal mice survive this dose of MRSA, we assume that the neutrophils (and macrophages) in the lung must be sufficient to ultimately clear the bacteria and protect the host.

In addition to neutrophils, anti-microbial peptides also contribute to killing of MRSA. Reg3γ has been demonstrated to have bacteriostatic activity against MRSA in vitro [30]. *In vivo*, Reg3γ is induced in the lungs following MRSA infection and inhibition with neutralizing anti-Reg3γ antibody decreased the clearance of MRSA in adult mice [30]. In our MRSA pneumonia model, we observed a lower level of Reg3γ mRNA expression in the neonate at baseline compared to the adult mice. However, both neonate and adult mice demonstrated an increase in expression of Reg3γ mRNA at 24hrs pi demonstrating that neonates are able to produce it (albeit at significantly lower levels). There was an increase in the expression of Reg3β in the neonate mice compared to the adult mice at 6 and 24 hr pi. These results suggest that the lack of Reg3γ in the neonatal lung prior to infection may contribute to the delayed clearance of MRSA in the neonates.

The neonatal lung environment is anti-inflammatory with increased levels of TGFβ and IL10 [36, 37]. In addition, the response to TLR stimulation in neonates is altered compared to adult cells with neonates producing less IFNγ and TNFα in response to TLR stimulation [22]. However, our studies demonstrate that neonates respond to MRSA infection with the expression of a cytokine profile similar to that of adult mice suggesting that there is a quantitative difference but not a qualitative difference between adults and neonates. The neonatal (and adult) mice in our study had an increase in cytokine mRNA expression for TNFα and IL17 by 2 hr pi which began to decline by 24 hr pi. Whereas IFNγ expression in both the neonates and adults was detectable at 6 hr pi and continued to increase at 24hr. IL17 is a critical cytokine for protection of mucosal sites against extracellular bacteria. It stimulates production of neutrophil chemokines and neutrophil growth and survival factors such as G-CSF and GM-CSF and production of anti-microbial peptides by epithelial cells, both of which are critical for clearance of extracellular bacteria [38, 39]. Individuals with a genetic deficiency in IL17 components exhibit increased *S*. *aureus* infections suggesting it is important in defense against this pathogen [40]. Our results demonstrate that neonatal mice were able to induce an IL17 response quickly after infection suggesting that it is not a deficiency in IL17 that is preventing efficient MRSA clearance in the neonatal lung.

The increase in TNFα and IFNγ mRNA in the neonatal lungs after MRSA infection demonstrate that neonates are capable of inducing a pro-inflammatory response following infection. We cannot differentiate whether the increase in pro-inflammatory cytokines is due to TLR stimulation of lung cells or the result of superantigen stimulation of resident neonatal T cells by one of the MRSA enterotoxins. Although others have suggested that TLR stimulation of neonatal cells results in a qualitatively different cytokine profile than adult cells, these studies were performed with neonatal cord blood and there may be distinct differences between the responses of these cells and innate immune cells in the neonatal lung.

The development of adaptive immune responses is dependent upon the presence of antigen presenting cells to dictate whether naïve CD4^+^ T cells differentiate into Th1, Th2 or Th17 type cells. Our studies revealed that there was no increase in the numbers of mDCs in the neonates following MRSA infection in comparison to the adults. In addition, the neonatal mDCs did not upregulate expression of CD86 in response to infection as was observed with the adult mDCs. The markers used to identify mDCs are shared with alveolar macrophages (AMs); and thus, we cannot rule out the presence of AMs in this population that we call mDCs. DCs are critical for determining the outcome of the adaptive immune response based on the profile of cytokines that they produce. Several studies have suggested that deficiencies in neonatal DCs results in the development of predominantly Th2 or Th17 type responses in the neonates [41, 42]. However, in our study the neonatal mice did not develop a Th2 response following MRSA infection. In addition, there was no significant induction of Th1 or Th17 cells in the neonates as compared to the adult mice following MRSA infection. Protective immunity to *S*. *aureus* is limited despite the observation of an adaptive immune response; individuals may become chronically infected or reinfected later in life [43, 44]. However, it is unlikely that the deficient T cell response in the neonatal mice contributes to disease severity in this model

One limitation of our study is the use of the same dose of MRSA for both the adult and neonatal mice. However, our goal was to determine if there were qualitative differences in the neonatal response to MRSA, for instance the development of an overwhelming Th2 response. The dose we chose was sublethal to allow us to examine the response in the absence of an overwhelming neutrophil response as well as to mimic natural exposures more closely.

Overall, we have developed a neonatal model of MRSA pneumonia that displays a similar delay in bacterial clearance as is observed in the NICU. This model will ultimately be used to determine the extent to which RSV coinfection contributes to the severity of MRSA pneumonia, similar to what is observed with influenza coinfection.

## Supporting Information

S1 Table Primer information for qRT-PCR of cytokines and anti-microbial peptides. (PDF)
