# Supplementary material for: A Neonatal Murine Model of MRSA Pneumonia
Source: PLoS One. 2017 Jan 6;12(1):e0169273. doi: 10.1371/journal.pone.0169273 (PMC5218573; doi:10.1371/journal.pone.0169273)
Supplement: S1 Table — (PDF) [file pone.0169273.s001.pdf]

S1 Table. Primer information for qRT-PCR of cytokines and anti-microbial peptides.

Gene	Forward Primer (5'-3')	Reverse Primer (5'-3')
Tnfα	TCTTCTCATTCCTGCTTGTGG	GGTCTGGGCCATAGAACTGA
Il6	ATGGATGCTGCTACCAAACTGGAT	CCAGGTAGCTATGGTACTCCAGA
Il17	TGTGAAGGTCAACCTCAAAGTCT	GAGGGATATCTATCAGGGTCTTCAT
Ifnγ	GGAGGAACTGGCAAAAGGAT	TTCAAGACTTCAAAGAGTCTGAGG
Reg3β	TGGATTGGGCTCCATGAC	TCATCACGTCATTGTTACTCCA
Reg3γ	ACCATCACCATCATGTCCTG	GGGCATCTTTCTTGGCAAC
